# Analysis and predication of tuberculosis registration rates in Henan Province, China: an exponential smoothing model study

**DOI:** 10.1186/s40249-020-00742-y

**Published:** 2020-08-31

**Authors:** Yan-Qiu Zhang, Xin-Xu Li, Wei-Bin Li, Jian-Guo Jiang, Guo-Long Zhang, Yan Zhuang, Ji-Ying Xu, Jie Shi, Ding-Yong Sun

**Affiliations:** 1 Henan Center for Disease Control and Prevention, Zhengzhou, 450016 P. R. China; 2 Center for Drug Evaluation, National Medical Products Administration, Beijing, 100022 P. R. China; 3 Kaifeng Municipal Health Commission, Kaifeng, 475000 P. R. China

**Keywords:** Active pulmonary tuberculosis, Registration rate, Prediction, Exponential smoothing model, Seasonality

## Abstract

**Background:**

The World Health Organization End TB Strategy meant that compared with 2015 baseline, the reduction in pulmonary tuberculosis (PTB) incidence should be 20 and 50% in 2020 and 2025, respectively. The case number of PTB in China accounted for 9% of the global total in 2018, which ranked the second high in the world. From 2007 to 2019, 854 672 active PTB cases were registered and treated in Henan Province, China. This study was to assess whether the WHO milestones could be achieved in Henan Province.

**Methods:**

The active PTB numbers in Henan Province from 2007 to 2019, registered in Chinese Tuberculosis Information Management System were analyzed to predict the active PTB registration rates in 2020 and 2025, which is conductive to early response measures to ensure the achievement of the WHO milestones. The time series model was created by monthly active PTB registration rates from 2007 to 2016, and the optimal model was verified by data from 2017 to 2019. The Ljung-Box Q statistic was used to evaluate the model. The statistically significant level is α = 0.05. Monthly active PTB registration rates and 95% confidence interval (*CI*) from 2020 to 2025 were predicted.

**Results:**

High active PTB registration rates in March, April, May and June showed the seasonal variations. The exponential smoothing winter’s multiplication model was selected as the best-fitting model. The predicted values were approximately consistent with the observed ones from 2017 to 2019. The annual active PTB registration rates were predicted as 49.1 (95% *CI*: 36.2–62.0) per 100 000 population and 34.4 (95% *CI*: 18.6–50.2) per 100 000 population in 2020 and 2025, respectively. Compared with the active PTB registration rate in 2015, the reduction will reach 23.7% (95% *CI*, 3.2–44.1%) and 46.8% (95% *CI*, 21.4–72.1%) in 2020 and 2025, respectively.

**Conclusions:**

The high active PTB registration rates in spring and early summer indicate that high risk of tuberculosis infection in late autumn and winter in Henan Province. Without regard to the *CI*, the first milestone of WHO End TB Strategy in 2020 will be achieved. However, the second milestone in 2025 will not be easily achieved unless there are early response measures in Henan Province, China.

## Background

Tuberculosis (TB) is a communicable disease that is a major cause of ill health, one of the top 10 causes of death worldwide. The case number of pulmonary TB (PTB) in China ranks the second high in the world, with an estimated incidence rate of 61/100 000 (range from 52/100 000 to 70/100 000) in 2018 [1]. PTB is also one of the major infectious diseases in Henan Province, China, where the resident population was 96.05 million in 2018 [2]. From 2007 to 2019, 854 672 active PTB cases were registered and treated in Henan Province [3], accounting for 7.6% of the countrywide [1]. The annual case number of PTB ranks the second high in infectious diseases in Henan Province [4].

From 2007 to 2019, Henan Province successively implemented the Stop TB Strategy and the End TB Strategy of the World Health Organization (WHO) [5, 6], and carried out many measures according to three TB prevention and control programs issued by the provincial government [3, 7, 8]. The main measures included providing free diagnostic services such as sputum smear and chest X-ray for TB suspects [7], providing TB patients with free basic anti-TB drugs, encouraging application of sputum smear, sputum culture and molecular biological methods to detect TB, carrying out health promotion, etc. [3]. However, the effect of TB prevention and control has not been systematically evaluated.

Using mathematical models to explore the pattern of incidence had been developed in infectious diseases control. For example, time series analysis was used for hand, foot, and mouth disease and TB [9, 10], autoregressive integrated moving average (ARIMA) model for hepatitis A and influenza [11, 12], temporal analysis for TB and human immunodeficiency virus (TB/HIV) co-infection [13]. Different models are suitable for different data characteristics. To find the most suitable model for active PTB registration rates from 2007 to 2019 in Henan Province, the SPSS software was used, which can automatically select the best-fitting model according to the time series data [14].

The WHO End TB Strategy means that compared with 2015 baseline, the reduction of PTB incidence should be 20 and 50% in 2020 and 2025, respectively [6]. In order to assess whether the WHO milestones could be achieved in Henan Province, time series analysis based on exponential smoothing (ES) model was used to predicate the active PTB registration rates in 2020 and 2025.

## Methods

### Data source

The active PTB numbers registered from 2007 to 2019 in Henan Province were extracted from the Chinese Tuberculosis Information Management System (CTIMS) [15]. The definition of active PTB was according to *the Health Standard of the People’s Republic of China WS196–2017* [16]. The statistical tables were derived by month. The numbers of residents in Henan Province from 2006 to 2018 were obtained from *Henan Statistical Yearbook* [2]. Assuming that the number of populations stayed unchanged during the year, the monthly and annual active PTB registration rates in Henan Province were calculated by the average number of populations at the middle of the years.

### Data analysis

This study was based on the active PTB registration rates in the whole province, and no personal information was involved. SPSS version 23.0 (SPSS, IBM; Inc., Chicago, IL, USA) was used for analysis and the statistically significant level is α = 0.05.

### Variables setting

Two variables, time and monthly registration rate, were set. Time series of monthly registration rates from January 2007 to December 2019 were input into SPSS software.

### Modeling and prediction process

We drew the time series diagram first. When there was a trend term or a period term, made a difference to the original data until it was nearly stable.

The second step was to calculate the autocorrelation function (ACF), partial correlation function (PACF) and the cross-correlation function (CCF) of the sample. Autocorrelation diagram and cross-correlation diagram were used to describe the characteristics of time series, and then difference and transformation were carried out. The peak registration rates of active PTB were judged by seasonal decomposition.

The third step was that all models were calculated by expert modeler module in traditional model of SPSS. Seasonal model was considered at the same time. The monthly activity PTB registration rates from 2007 to 2016 were used to fit the time series model.

The model was verified by monthly active PTB registration rates from 2017 to 2019, and then predicted the monthly rates from 2020 to 2025. Based on the predicted monthly rates, the annual rates were calculated.

### Model evaluation

The Expert Modeler module in SPSS can automatically filter the best-fitting model according to the set conditions.

The goodness of fitting was measured by stationary R-squared. The Ljung-Box Q statistic was used to evaluate whether the model was correctly specified. Mean absolute percentage error (MAPE) was utilized to test the accuracy. When MAPE is less than or equal to 10%, it means highly accurate forecast [17]. The forecast ability of the model was tested by predicting the monthly active PTB registered rates from 2017 to 2019. The model was used to predict the active PTB registration rates from 2020 to 2025.

## Results

### The characteristics of registration rates

From 2007 to 2019, the active PTB registration rates in Henan Province showed a decreasing trend from 88.0/100 000 to 49.0/100 000 in Table 1. According to the formula of average development rate [18], the average development rate of active PTB registration rates in 13 years was 95.2%, that is, the annual decline of registration rates was 4.8%.

**Table 1:** Monthly and annual active PTB registration rates from 2007 to 2019 in Henan Province, China

Year	Registration rate (per 100 000)													Population
	Jan.	Feb.	Mar.	Apr.	May	Jun.	Jul.	Aug.	Sept.	Oct.	Nov.	Dec.	Annual	(100 000)
2007	7.6	5.6	9	9.2	8.5	8	7.4	7.6	6.8	6.5	6.8	5	88.0	937.6
2008	6.3	5.9	9.3	9.4	8.6	8.2	7.2	6.8	6.5	7.2	6.9	5.9	88.0	939.5
2009	5	6.5	8.5	8.3	7.5	7.8	6.6	6.6	6.5	6.4	6	6.3	82.1	945.8
2010	6	4.9	7.4	7.3	7.3	7.3	6.3	6	5.7	5.7	6.3	5.7	75.9	944.6
2011	5.2	5	7.1	6.7	6.9	6.5	5.8	5.8	5.3	5.4	6.3	5.9	71.9	939.7
2012	4.6	6.6	7.4	7.3	7.1	6.6	5.8	5.9	5.6	5.7	5.8	5.5	73.9	939.7
2013	5.1	4.7	6.8	6.1	6.5	5.9	5.5	5.3	5.5	5.7	5.6	5.7	68.4	941.0
2014	5.2	4.6	6.4	6.3	6.1	6	5.4	5.1	5	5.2	5.6	6.3	67.4	942.5
2015	5	4.1	6.2	5.9	5.6	5.8	5.3	5.2	5.3	4.9	5.2	5.7	64.3	945.8
2016	4.5	4.4	5.9	5.6	5.4	5.3	4.9	5.1	4.9	4.5	5.1	5.2	60.7	950.6
2017	3.8	4.9	5.6	5.2	5.3	5.4	4.6	4.9	4.8	4.4	5	5	58.8	954.6
2018	4	3.8	5.8	5	5.4	5.1	4.5	4.6	4.5	4.3	4.6	4.5	55.9	958.2
2019	4.2	3.6	5.1	4.8	4.5	4.4	4.4	3.8	3.7	3.2	3.6	3.7	49.0	962.3

*PTB* Pulmonary tuberculosis

### Time series analysis

**Fig. 1:**
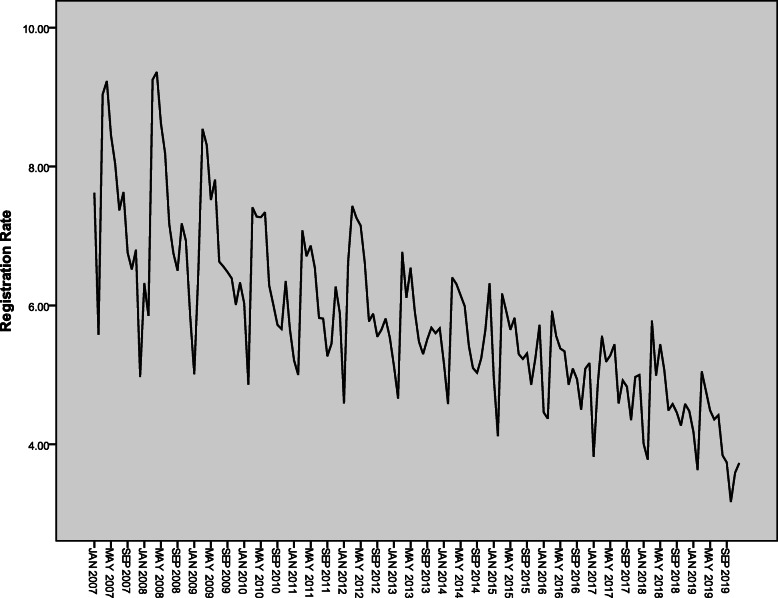
Time sequence of active PTB registration rates from 2007 to 2019 in Henan Province, China (1/100 000). PTB: Pulmonary tuberculosis

**Fig. 2:**
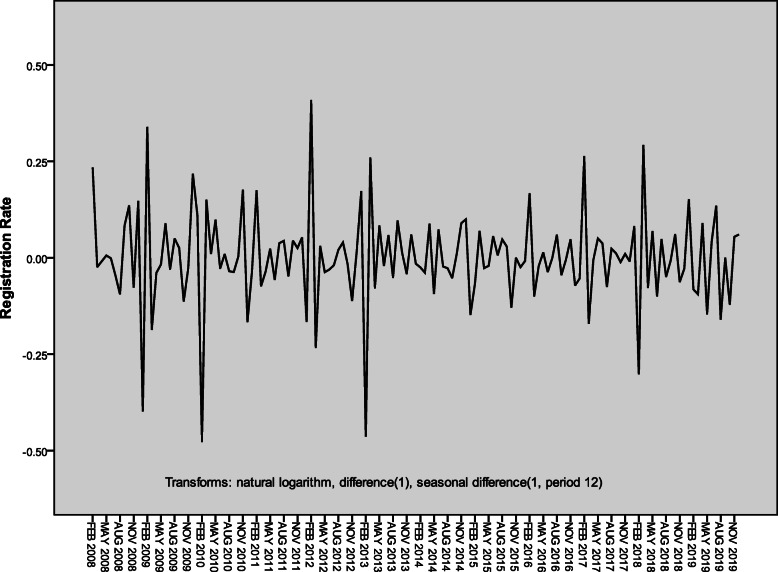
Time sequence of active PTB registration rates from 2007 to 2019 after transforming and differencing in Henan Province, China (1/100 000). PTB: Pulmonary tuberculosis

The monthly active PTB registration rates from 2007 to 2019 in Henan Province showed a trend of volatility and decline (Fig. 1). By differences and transformation including one order difference, one order seasonal difference and the natural log (LN) transformation, the time series showed the stationary (Fig. 2). It conformed to the requirement of the time series analysis.

**Fig. 3:**
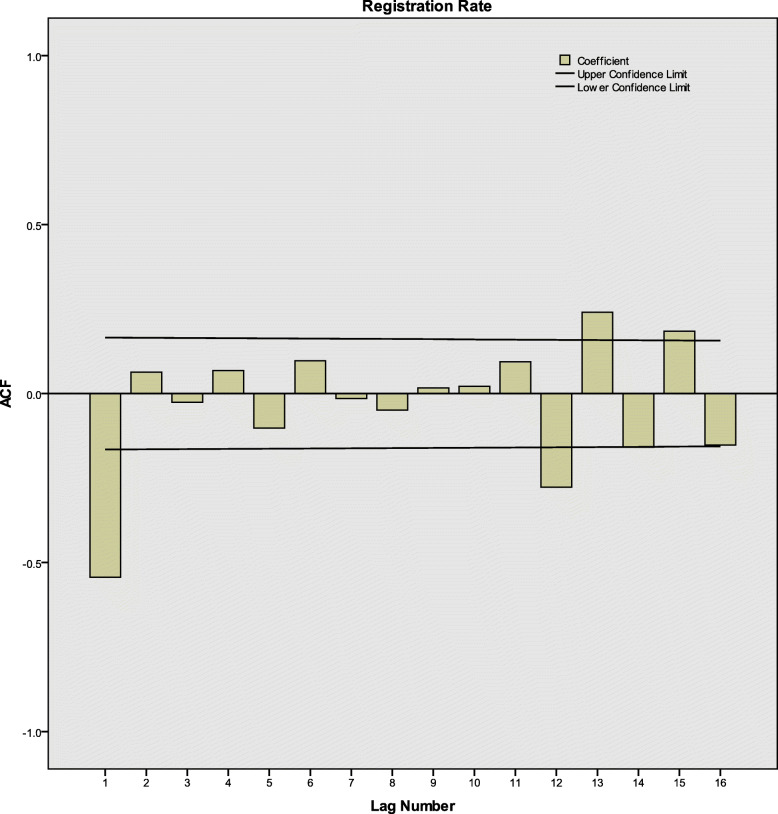
ACF of the monthly active PTB registration rates in Henan Province, China. ACF: Autocorrelation function; PTB: Pulmonary tuberculosis

**Fig. 4:**
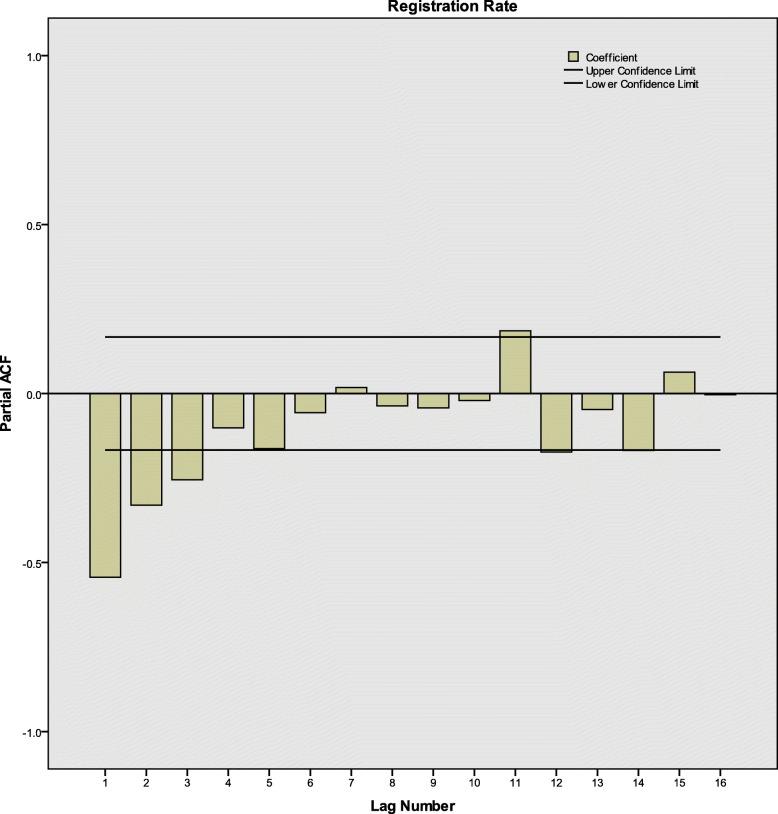
PACF of the monthly active PTB registration rates in Henan Province, China. PTB: Pulmonary tuberculosis; PACF: Partial autocorrelation function

**Fig. 5:**
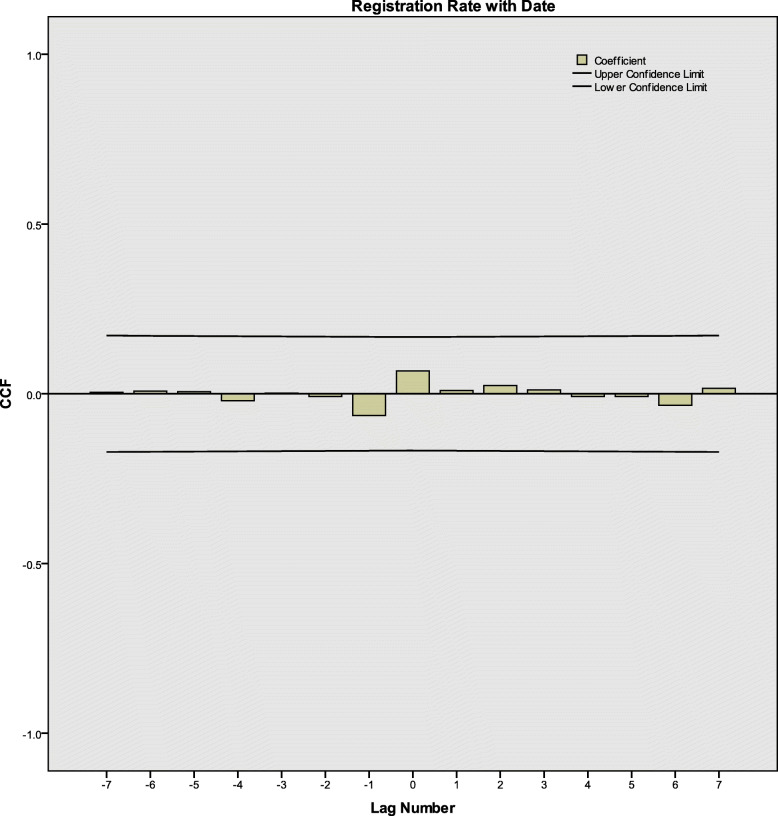
CCF of active PTB registration rates and time in Henan Province, China. PTB: Pulmonary tuberculosis; CCF: Cross correlation function

After differences and transformation, according to ACF, PACF and CCF diagrams (Fig. 3, 4 and 5), there were neither correlation between the registration rates nor between registration rates and time. The series was white noise.

Through seasonal decomposition, we got the seasonal factors in each month (Table 2). March, April, May and June accounted for high active PTB registration rates.

**Table 2:** Seasonal factors (%) for the active PTB registration rates from 2007 to 2019 in Henan Province, China

Month	Jan.	Feb.	Mar.	Apr.	May	Jun.	Jul.	Aug.	Sept.	Oct.	Nov.	Dec.
Seasonal factors	86.6	83.0	117.9	113.6	112.5	109.7	97.9	95.9	93.1	94.2	99.3	96.3

*PTB* Pulmonary tuberculosis

### Selection of the model

Through the Expert Modeler, the exponential smoothing (ES) winters multiplication model was selected as the best-fitting model. By one order difference, one order seasonal difference and LN transformation, the model fit statistics were shown in Table 3.

**Table 3:** Exponential smoothing model fitting for the active PTB registration rates from 2007 to 2016 in Henan Province, China

Model	Stationary R-squared	R-squared	RMSE	MAPE	MAE	MaxAPE	MaxAE	Normalized BIC
Registration Rate	0.606	0.839	0.452	5.527	0.334	28.253	1.742	-1.469

*PTB* Pulmonary tuberculosis; *RMSE* Root mean square error; *MAPE* Mean absolute percentage error; *MAE* Mean absolute error; *MaxAPE* Max absolute percentage error; *MaxAE* Max absolute error; *BIC* Bayesian Information Criterion

Because the dependent variable data were seasonal data, the Stationary R-squared was more representative. The Stationary R-squared of the model was 0.606, the R-squared was 0.839, and the normalized Bayesian Information Criterion (BIC) was -1.469, which showed that the fitting of the model was good. The MAPE of the model was 5.527%, which indicated that the forecast effect was good. The residual sequence was tested by white noise (Ljung-Box Q [18] = 11.862, *P* = 0.689). Therefore, the hypothesis based on the independent residual sequence was acceptable. The model had already fully extracted information. It was suitable for the ES model to be used for the prediction.

Of the three parameters of the fitting model, the seasonal parameter (Delta) had statistical significance (*P* = 0.000), and the stationary parameter (Alpha) and the trend parameter (Gamma) of time series had no statistical significance (*P* = 0.796 and *P* = 0.996, respectively), indicating that there was no horizontal and linear trend in this time series.

### Validity of the model

**Fig. 6:**
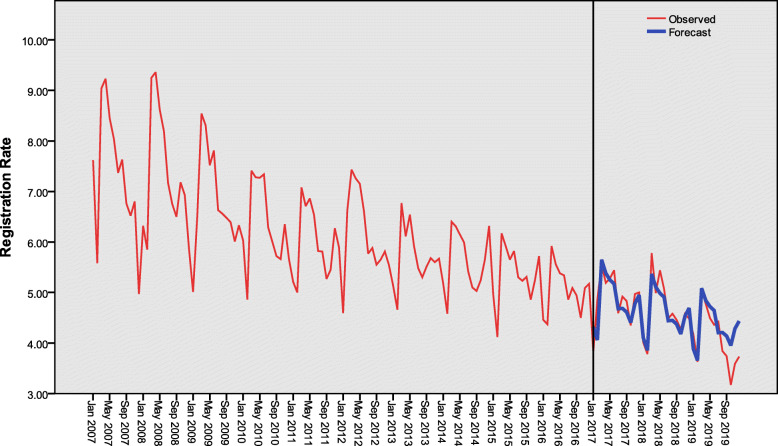
Comparison between the observed and predicted values of active PTB registration rates from 2017 to 2019 in Henan Province, China (1/100 000). PTB: Pulmonary tuberculosis

According to the established ES model, the predicted values of monthly active PTB registration rates in Henan Province were replace by the observed ones from 2017 to 2019. The mean absolute error (MAE) was 0.334%. The predicted values were basically consistent with the observed ones (Fig. 6).

### Prediction for 2020 and 2025

**Fig. 7:**
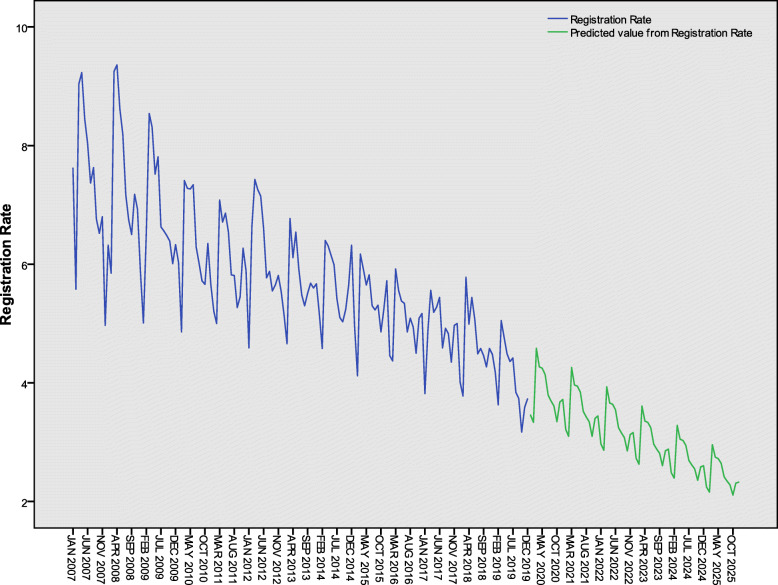
Time series analysis and prediction for active PTB registration rates from 2007 to 2030 in Henan Province, China (1/100 000). PTB: Pulmonary tuberculosis

The ES model was applied to predict monthly and annual active PTB registration rates from 2020 to 2025 in Henan Province. The predicted values of the annual registration rates can be seen in Table 4. The annual active PTB registration rates were 49.1 (95% *CI*: 36.2–62.0) and 34.4 (95% *CI*: 18.6–50.2) per 100 000 population in 2020 and 2025, respectively. The fitting and forecast results of monthly active PTB registration rates were shown in Fig. 7. Compared with the active PTB registration rate in 2015, the reduction will be 23.7% (95% *CI*: 3.2–44.1%) and 46.8% (95% *CI*: 21.4–72.1%) in 2020 and 2025, respectively.

**Table 4:** The predicted annual active PTB registration rates from 2020 to 2025 in Henan Province, China (1/100 000)

	2020	2021	2022	2023	2024	2025
Predicted	49.1	46.2	43.2	40.3	37.3	34.4
95% UCL	62.0	59.7	57.4	55.0	52.6	50.2
95% LCL	36.2	32.6	29.1	25.5	22.0	18.6

*95% UCL* 95% upper confidence limit; *95% LCL* 95% lower confidence limit

## Discussions

This study showed that the months of higher active PTB registration rates in Henan Province were March, April, May, June. The ES model indicated that there were significant seasonal variations. The similar results were found at the national level and in other provinces. A study showed that from 2004 to 2008, April was the peak month for student TB cases in China, followed by May and March [19]. March to June is the period of physical examination for students in the middle school entrance examination and the college entrance examination, which means a screening for students. This factor may be one of the reasons for the high registration rates from March to June. The seasonality of active TB registration was peaked in March in Xinjiang, China [20]. There were also some studies on the seasonality and trend analysis of TB incidence around the world [21–24]. From 1993 to 2008, 21.4% cases were diagnosed in March, the peak month in the US [25].

A study in Singapore believed that the ARIMA model was effective in predicting the short-term trend of TB [26]. Zhang et al. [27] used seasonal ES model to predict the number of PTB cases in Shenzhen, in which the Stationary R-squared was 0.68 and the Ljung-Box Q statistic *P* value was 0.86. Its parameters were close to that of the ES model in this study. The ES model is a time series analysis method developed on the basis of the moving average model [28]. The ES value of any period is the weighted average of the actual observed value in the current period and the previous value. The ES model does not abandon the previous data, but gradually reduces the weight of the previous data.

Ríos et al. [29] from Spain thought that the tubercle bacilli expelled from infected persons in a room with closed windows may increase the risk of exposure of healthy persons in winter and the clinical onset would be in spring. According to this, we thought that the seasonal peak in March in Henan Province may be related to the Spring Festival holiday. During the Spring Festival, all the family members gather together to celebrate and seeing a doctor when feeling ill is a taboo. The closed windows in winter, large-scale mobilization, and health-seeking delay would jointly result in the increase and accumulation of PTB cases after the Spring Festival holiday, often in March. On World TB Day every year, many activities were organized in Henan to promote tuberculosis knowledge. This raises public awareness of TB, leading to seeking medical advice. This is one reason why the registration rates increased after March.

Globally, the average decline rate of the TB incidence was 1.6% per year during 2000 to 2018 [1]. From annual TB reports of the WHO [1], we can get Chinese annual TB registration rates from 2007 to 2018. The annual decline rate was 2.29% in China. From 2007 to 2019, the active PTB registration rate decreased from 88.0/100 000 to 49.0/100 000 with a 4.8% annual decline in Henan Province. Overall, the decline of incidence rate in Henan Province is greater than that in nationwide and worldwide. Du et al. [30] thought that the decline of TB incidence and prevalence was related to economic development in China. Apart from economic development, we thought that it was related to the application of molecular biological diagnosis in Henan Province in recent years, so that patients can be diagnosed and treated in time.

The hypothesis of time series analysis is based on the principle of inertia, that is, under certain conditions, the past trend of the predicted things will continue to the future. The ES model gives larger weight to recent observation values and gives smaller weight to earlier ones. In accordance with the decline trend in recent years, without the adoption of new measures, the predicted active PTB registration rate will reach 49.1 (95% *CI*: 36.2–62.0) per 100 000 population in 2020 and 34.4 (95% *CI*: 18.6–50.2) per 100 000 population in 2025 in Henan Province. Compared with the active PTB registration rate in 2015 (64.3/100 000), the reduction will be 23.7% (95% *CI*: 3.2–44.1%) and 46.8% (95% *CI*: 21.4–72.1%) in 2020 and 2025, respectively.

The missing report rate of infectious disease in medical institutions was 3.18% in 2012 in Henan Province, top two were syphilis and TB [31]. Assuming that the missing report rate of active PTB unchanged and keeping the TB control strategy remain unchanged in 2020 in Henan Province, without regard to the *CI*, the first milestone (20% reduction) of WHO End TB Strategy in 2020 will be achieved.

The point prediction in 2025 was 34.4 per 100 000 population and it had a large range from 18.6 to 50.2 per 100 000 population. So, to achieve the second WHO milestone, new measures must be taken. In order to improve the diagnosis [32–35], treatment [36, 37] and TB prevention services [38–40], a lot of research have been carried out around the world. A study from Nepal found that active case finding could reduce catastrophic costs [41]. And the WHO milestones can only be achieved within the context of progress towards universal health coverage (UHC) [1]. In 2018, the policy of PTB diagnosis related groups based payment (DRGs) was launched in Henan Province [42]. Patients only need to bear 20% of the fixed cost based on different clinical pathway. This financing policy will help to improve patient’s treatment compliance. The End TB Strategy encompasses a package of interventions that fall under three pillars [6]. Since 2020, the establishment of an electronic information system for hospitals, Centers for Disease Control and Prevention and primary health institutions will be explored to close gaps between incidence and notification in Henan Province. We will try to establish an infection control model based on primary health institutions to reduce the chance of infection in close contacts as well. We will carry out active screening of key populations and get multi-drug resistant TB (MDR-TB) patients timely diagnosed and treated. In 2015, the public total awareness rate of TB core information in Henan Province was 72.1% [43], so we need to strengthen public health education. We hope that with our efforts, the second WHO milestone objective will be achieved in 2025 in Henan Province.

The limitations of the study should be acknowledged. Only 13 years of registration data were obtained and analyzed because the CTIMS was established in 2004. The relatively short length of the series may influence the forecasting efficacy. The predictive effect of long term forecast by the time series may be weak because of the uncontrollable of the change of the factors. Although seasonal variation in TB incidence has been described in several recent studies, the mechanism underlying this seasonality remains unknown. Next, we will conduct further study to describe patterns of seasonality in active PTB population with different characteristics and try to find the reason of seasonality.

## Conclusions

The high active PTB registration rates in spring and early summer indicates that high risk of TB infection in late autumn and winter in Henan Province. Without regard to the *CI*, under the premise that the whole TB control environment does not change, the first milestone of WHO End TB Strategy in 2020 will be achieved. However, based on the predicted active PTB registration rates, the second milestone in 2025 will not be easily achieved unless there are early response measures in Henan Province, China. Since 2018, some new measures, such as UHC was implemented in hoping to achieve the second WHO milestone objective in 2025 in Henan Province.

## Data Availability

All data generated or analyzed during this study are included in this published article.

## Abbreviations

TB: Tuberculosis
PTB: Pulmonary tuberculosis
WHO: World Health Organization
CTIMS: Chinese Tuberculosis Information Management System
95% *CI*: 95% confidence interval
ES model: Exponential smoothing model
ARIMA: Autoregressive integrated moving average
TB/HIV: Human immunodeficiency virus
ACF: Autocorrelation function
PACF: Partial correlation function
CCF: Cross-correlation function
MAPE: Mean absolute percentage error
LN: Natural log
Normalized BIC: Normalized Bayesian Information Criterion
MAE: Mean absolute error
UHC: Universal health coverage
DRGs: Diagnosis related groups based payment
MDR-TB: Multi-drug resistant TB
